# Experimental and Theoretical Investigation into the Thermal Conductivity and Heating-Softening Bending of Glass-Fiber-Reinforced Polypropylene Rebars

**DOI:** 10.3390/polym17050595

**Published:** 2025-02-24

**Authors:** Mingxue Xu, Anni Wang, Xiaogang Liu

**Affiliations:** Research Institute of Urbanization and Urban Safety, School of Future Cities, University of Science and Technology Beijing, Beijing 100083, China; xumingxue1991@163.com (M.X.); anniwang@ustb.edu.cn (A.W.)

**Keywords:** glass fiber, polypropylene, thermoplastic, thermal conductivity, heating-softening processing time

## Abstract

Thermoplastic fiber-reinforced polymer (FRP) reinforcement has a significant advantage over traditional thermosetting FRP reinforcements in that it can be bent on site by heating-softening processing. However, current experimental and theoretical research on the thermal conductivity and heating-softening processing characteristics of thermoplastic FRP reinforcements is quite insufficient. Through heating-softening processing tests, numerical simulation, and theoretical calculation, this study investigated the heating-softening processing time of a thermoplastic glass-fiber-reinforced polypropylene (GFRPP) reinforcement. In the heat transfer process, thermal conductivity is typically treated as a constant. However, the experimental results indicated that the thermal conductivity/diffusivity coefficient of the GFRPP reinforcement was temperature-dependent. On this basis, an equivalent modified thermal diffusivity coefficient of glass fiber was proposed to account for the time-temperature-dependent heat conductivity of the GFRPP reinforcement, utilizing a series model. Utilizing the modified thermal diffusivity coefficient, the simulation model presented a heating-softening processing time that coincided well with the experimental results, with a mean ratio of 1.005 and a coefficient of variation of 0.033. Moreover, based on an equivalent homogeneous circular cross-section assumption of the GFRPP reinforcement, an analytical solution to the heat conduction equation was derived. Combining the experimental and simulation results, a semi-analytical and semi-empirical calculation model was also proposed for predicting the heating-softening processing time of a GFRPP reinforcement with a silicone tube cover. The model’s calculated results align with the simulation trends, with an average deviation of 1.0% and a coefficient of variation of 0.026, demonstrating strong potential for engineering applications.

## 1. Introduction

Reinforced concrete structures are widely used in civil engineering due to their low cost, good integrity, and ease of construction [1]. However, traditional reinforced concrete has poor crack resistance and generally works with cracking, which creates channels for material exchange between the internal rebars and the external environment, thus inducing rebar corrosion, especially in marine corrosive environments. This corrosion not only weakens the mechanical properties of rebars, but also accelerates the deterioration of the concrete, thereby shortening the structure’s service life [2,3,4,5,6]. Although concrete cracks can be relieved through prestressing technology, this significantly increases construction costs, and the corrosion and prestressing relaxation of the prestressed tendons cannot be fully overcome. Therefore, fiber-reinforced polymer (FRP) reinforcements with excellent chloride corrosion resistance have been increasingly widely used in pavement and bridge engineering to replace traditional rebars [7].

Generally, FRP reinforcements can be categorized into carbon-fiber-reinforced polymers (CFRPs), aramid-fiber-reinforced polymers (AFRPs), and glass-fiber-reinforced polymers (GFRPs), among which GFRP reinforcements are the most widely used in engineering applications due to their higher cost performance. GFRP reinforcements can also be divided into thermoplastic and thermosetting types, distinguished by the resin matrix [8,9]. Currently, due to mature production technology and the supply system, thermosetting FRP reinforcements are the most widely used; however, they are unable to be reprocessed or bent in situ, causing inconvenience for engineering construction. In contrast, thermoplastic FRP reinforcements allow for on-site processing due to their heating-softening characteristics, making the production of stirrups and hook handling more convenient [10]. Additionally, thermoplastic FRPs also exhibit higher ductility and impact resistance [11,12,13,14,15,16], which is beneficial for structural mechanical performance. Therefore, thermoplastic FRP reinforcements have broad prospects in engineering applications. Thermoplastic resins generally include polyether ether ketone, polyamide, polyphenylene sulfide, polyethylene terephthalate, polymethyl methacrylate, and polypropylene [17,18,19,20,21,22]. Although their high viscosity affects impregnation efficiency and is prone to causing insufficient fiber-resin interface bonding, the utilization of modification technology has improved the impregnation performance of polymethyl methacrylate and polypropylene [23]. Due to the advantages of cost-effectiveness, easy processing, good mechanical performance, and the hydrothermal resistance of polypropylene, glass-fiber-reinforced polypropylene (GFRPP) reinforcements are attracting attention due to showing tensile, bending, and interlayer shear properties comparable to traditional thermosetting GFRP reinforcements [24].

Although thermoplastic GFRPP reinforcements can be repeatedly processed on-site by heating-softening, the heating-softening processing technique used is critical. The basis of heating-softening processing is heat conduction theory, which was first proposed by Joseph Fourier [25], who provided a quantitative description of heat transfer and defined the relationship between heat flux density and temperature gradient. Maxwell further developed the microscopic theory of heat conduction, integrating molecular dynamics theory to describe heat conduction phenomena in gases and solids [26]. Boltzmann further advanced its statistical mechanics, providing a microscopic mechanism for understanding heat conduction [27]. Debye and Born introduced phonon theory, explaining heat conduction as the propagation of phonons in a lattice, which deepened our understanding of heat conduction in solid materials [28,29]. Kapitza investigated heat conduction phenomena at low temperatures and proposed the interface thermal resistance theory, promoting an understanding of heat conduction behavior at the interfaces of different materials [30]. With the advancement of nanotechnology, research on heat conduction is no longer limited to macroscopic scales, and research on thermoelectric materials and nanomaterials has revealed new thermal conduction properties [31].

The mechanism of heat transfer in solids primarily occurs through charge carriers or phonons. In metals, the heat transfer is dominated by electrons, while in insulators and semiconductors, it is primarily governed by phonons. The main mechanism of heat transfer in polymers is also the phonon transfer, but phonons are prone to scattering during the transfer process [32]. According to relevant studies, the thermal conductivity of most polymers is low, making it difficult to meet the demands for high thermal conductivity applications [33]. To achieve higher thermal conductivity, researchers have introduced nanofillers, such as carbon nanotubes and graphene, into thermoplastic composites, analyzing the effects of filler type, concentration, and dispersion methods on thermal conductivity [34,35,36,37,38]. Existing research has also shown that the thermal conductivity of polymer composites depends on the structures and properties of both the polymers and the fillers, as well as the morphology of the composites and the interactions between the polymers and the fillers [39].

The thermal conductivity of thermoplastic glass-fiber-reinforced polypropylene (GFRPP) reinforcement is primarily influenced by the distribution of glass fibers (GFs) within the polypropylene (PP) resin and their thermal conductivity properties. However, few researchers have linked the thermal conductivity to the changes in the temperature field, which leads to the assumption that the thermal conductivity remains a constant value in the heat transfer process, rather than being a state-dependent parameter that varies with the temperature field.

Investigating the thermal conductivity and the heating-softening time of GFRPP reinforcements is crucial for their production, processing, and bending. Currently, experimental and theoretical research on the heat transfer mechanisms and heating-softening time of thermoplastic composites is relatively limited, and there is a lack of heating-softening models to guide the thermal processing of GFRPP reinforcements, necessitating further exploration. Based on existing GFRPP reinforcements, the thermal conductivity characteristics and heating-softening time were studied through experiments, numerical simulations, and theoretical calculations. Firstly, the heating-softening time of the GFRPP reinforcement at different heating temperatures was experimentally measured. Furthermore, using the Monte Carlo method to generate a cross-sectional model of the thermoplastic GFRPP reinforcement, a numerical simulation model was established. By comparing the simulation and experimental results, it was revealed that the thermal diffusivity coefficient of the GFRPP reinforcement exhibited significant time-temperature dependence. In response to this phenomenon, an equivalent modified thermal diffusivity coefficient for glass fibers was proposed based on a series model, to account for the time-temperature-dependent thermal conductivity of the GFRPP reinforcement, resulting in good agreement between the softening time of the simulation model and the experimental results. Additionally, based on the assumption of an equivalent homogeneous circular cross-section of the GFRPP reinforcement, an analytical solution to the heat conduction equation was derived. Combining the experimental and simulation results, a semi-analytical and semi-empirical calculation model was also proposed to predict the heating-softening processing time of the GFRPP reinforcement with a silicone tube cover, and its feasibility was validated through comparison with the simulation results.

## 2. Theory of Heat Conduction

### 2.1. Fourier’s Law

Heat conduction is the process of heat transfer from one object to another, relying on a temperature difference between the objects to facilitate it. In the absence of external work, the heat naturally flows from areas with higher temperatures to areas with lower temperatures. Fourier’s research found that the rate of heat conduction was proportional to the temperature gradient [25], as expressed by Equation (1).

(1) $$\vec{q}=-k\nabla T$$

where $\vec{q}$ represents the heat flux density on a unit area of the object, with a unit of W/m^2^; *k* is the material thermal conductivity, assessing the material heat conduct ability, with a unit of W/(m·K); and *T* is the temperature, with a unit of K.

### 2.2. Unsteady-State Heat Conduction Equation

During the heat conduction process in a system, if the temperature distribution *T* changes with time *t*, the system is in an unsteady heat conduction state. In this case, the time factor must be considered. According to the energy conservation principle [40], the heat flowing into the system should equal the heat absorbed by the object to raise its temperature. Thus, the general form of the three-dimensional heat conduction equation can be expressed as Equation (2).

(2) $$\frac{\partial T}{\partial t}=\frac{k}{c\rho }\nabla^{2}T+\frac{F\left(x,y,z,t\right)}{c\rho }$$

where *c* represents the specific heat capacity of materials, with a unit of kJ/(kg·K); *ρ* is the material density, with a unit of kg/m^3^; $F\left(x,y,z,t\right)$ denotes the heat source intensity within the object, which is zero since there is no heat source inside the GFRPP bar.

### 2.3. Analytical Method

#### 2.3.1. Homogeneity of the Material

In continuum mechanics, a uniform material is not necessarily homogeneous. Homogeneity is a stronger definition than uniformity, specifically manifested in that all particles of a uniform material are isomorphic to each other through a single reference configuration, and their properties are independent of the particles themselves [41]. This implies that there is only one property that characterizes the continuity of the material. The mathematical description of material homogeneity is that in the configuration space $\left(\Sigma ,X\right)$, $\exists x_{0}\in X$, such that within the neighborhood $B\left(x_{0},a\right)$ of a radius a around the point $x_{0}$, the property functional $\varphi \left(x\right)$ satisfies Equation (3). Therefore, it is said that the property functional $\varphi \left(x\right)$ exhibits homogeneity within ***B***. The parameters *c*, *ρ*, and *k* in the heat conduction Equation (2) are all property functionals, and Equation (2) can only be directly solved when the system satisfies material homogeneity.

(3) $$\varphi \left(x\right)=\varphi \left(x_{0}\right)$$

#### 2.3.2. Analytical Theory of Unsteady State Heat Conduction Within a Homogeneous Circular Plane

**Figure 1:**
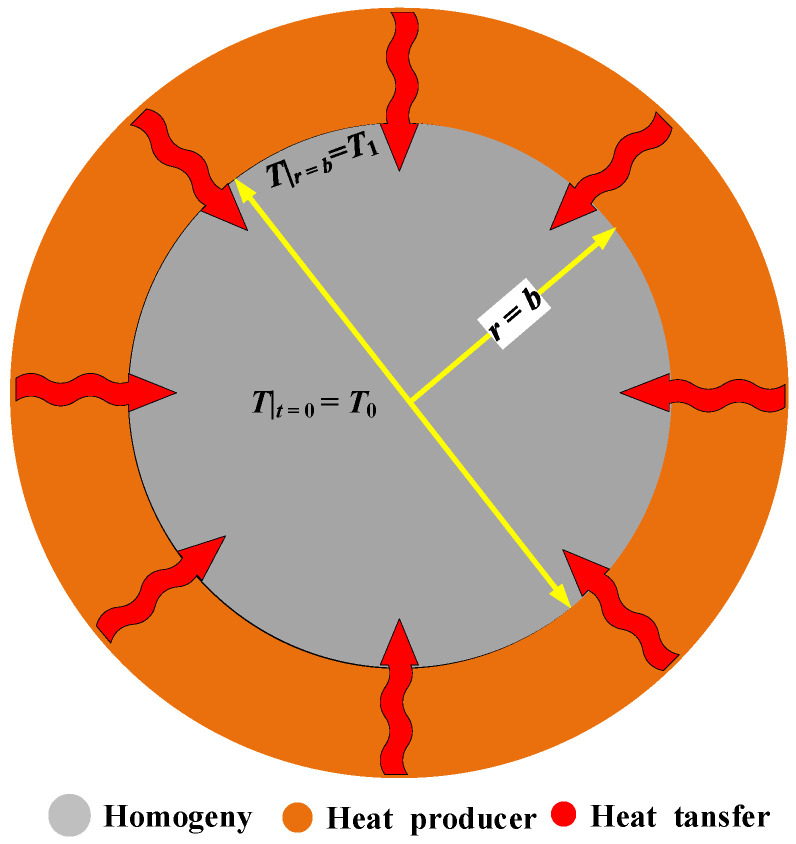
Homogeneous two-dimensional circular heat system.

The temperature distribution at time *t* in a two-dimensional homogeneous circular plane with a radius *b* is analyzed. As shown in Figure 1, the surrounding heat source continuously supplies heat to the boundary, maintaining a constant temperature *T*_1_ at the boundary of the circle. At the initial moment, the temperature inside the circular plane is *T*_0_. Given that there are no heat sources within the plane, the two-dimensional heat conduction Equation (4) can be derived from Equation (2).

(4) $$\frac{\partial T}{\partial t}=\frac{k}{c\rho }\left(\frac{\partial^{2}T}{\partial x^{2}}+\frac{\partial^{2}T}{\partial y^{2}}\right)$$

Based on the system being a circular plane, Equation (4) can be converted into the polar coordinate form, as Equation (5).

(5) $$\frac{\partial T}{\partial t}=\frac{k}{c\rho }\left(\frac{\partial^{2}T}{\partial r^{2}}+\frac{1}{r}\frac{\partial T}{\partial r}+\frac{1}{r^{2}}\frac{\partial^{2}T}{\partial \theta^{2}}\right)$$

Since the heat transfer occurs from the circumference to the interior, this problem also satisfies the axial symmetry. According to the axial symmetry, the temperature *T* is independent of the angle *θ* and is only a function of time *t* and radius *r*. Thus, Equation (5) simplifies to Equation (6).

(6) $$\frac{\partial T}{\partial t}=\frac{k}{c\rho }\left(\frac{\partial^{2}T}{\partial r^{2}}+\frac{1}{r}\frac{\partial T}{\partial r}\right)$$

The general solution of Equation (6) is given by Equation (7).

(7) $$T\left(r,t\right)=C+\sum_{n=1}^{+\infty }\left[{A_{n}J}_{0}\left(\sqrt{\lambda }r\right)+B_{n}Y_{0}\left(\sqrt{\lambda }r\right)\right]e^{-\lambda \frac{k}{c\rho }t}$$

where $\lambda ={\left[\frac{x_{n}^{\left(0\right)}}{b}\right]}^{2}$, 0<r<b, $x_{n}^{\left(0\right)}$ is the *n*th zero point of the zeroth-order Bessel function $J_{0}\left(x\right)$, and *C* is the constant. For GFRPP, the temperature is finite at *r* = 0 (the core region), so the coefficients of the second kind of Bessel series are zero. Therefore, Equation (7) simplifies to Equation (8).

(8) $$T\left(r,t\right)=C+\sum_{n=1}^{+\infty }{A_{n}J}_{0}\left(\sqrt{\lambda }r\right)e^{-\lambda \frac{k}{c\rho }t}$$

From the boundary condition $T{|}_{r=b}=T_{1}$, Equation (9) can be achieved from Equation (8).

(9) $$T\left(r,t\right)-T_{1}=\sum_{n=1}^{+\infty }{A_{n}J}_{0}\left(\sqrt{\lambda }r\right)e^{-\lambda \frac{k}{c\rho }t}$$

Substituting the initial condition $T{|}_{t=0}=T_{0}$ into Equation (9) and expanding the left side of the equation using Bessel functions allows for a comparison of the coefficients in the series on both sides of the equation, leading to Equation (10).

(10) $$A_{n}=\frac{2T_{0}-2T_{1}}{{\left[J_{0}^{\prime }\left(x_{n}^{\left(0\right)}\right)\right]}^{2}b^{2}}\int_{0}^{b}rJ_{0}\left(\frac{x_{n}^{\left(0\right)}}{b}r\right)dr$$

By solving Equation (10), the expression for the coefficient $A_{n}$ can be obtained as Equation (11).

(11) $$A_{n}=\frac{2T_{0}-2T_{1}}{J_{1}\left(x_{n}^{\left(0\right)}\right)x_{n}^{\left(0\right)}}$$

Substituting Equation (11) into Equation (9) allows for the solution of the homogeneous circular plane heat conduction equation, as in Equation (12).

(12) $$T\left(r,t\right)=T_{1}-2\left(T_{1}-T_{0}\right)\sum_{n=1}^{+\infty }{\frac{1}{J_{1}\left(x_{n}^{\left(0\right)}\right)x_{n}^{\left(0\right)}}J}_{0}\left(\frac{x_{n}^{\left(0\right)}}{b}r\right)e^{-{\left[\frac{x_{n}^{\left(0\right)}}{b}\right]}^{2}\frac{k}{c\rho }t}$$

#### 2.3.3. Equivalent Homogenization of Property Functional

**Figure 2:**
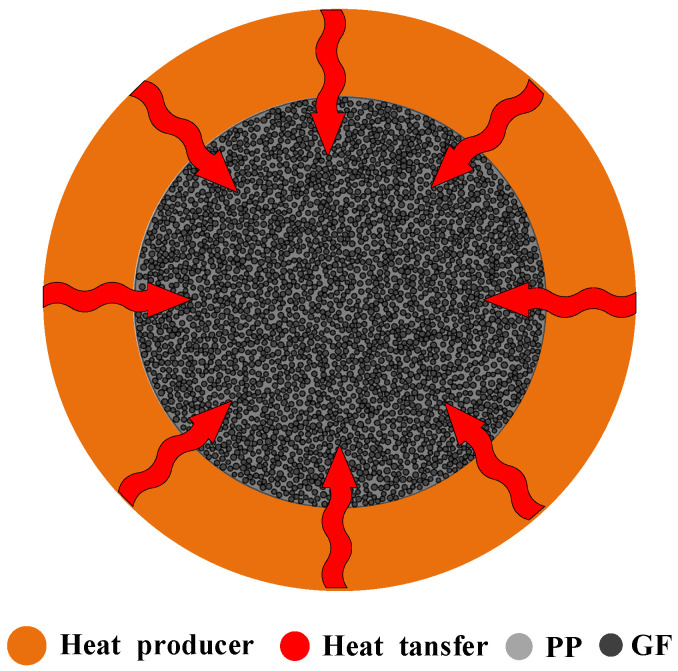
GFRPP reinforcement two-dimensional circular heat system.

The heated system of the GFRPP circular cross-section, as illustrated in Figure 2, features a uniform doping characteristic, with glass fibers (GFs) evenly dispersed within the polypropylene (PP) resin matrix. GFs and PP represent two distinct components that exhibit significantly different physical properties. To apply homogenization theory, it is necessary to perform property equivalency treatment for this system, which requires consideration of the distribution and arrangement of GFs and PP in this system. The basic calculation models for the property equivalency primarily include the parallel model and the series model [39].

In the series model, the various components (GFs and PP) in the system are treated as independent parts connected in sequence. The overall performance of the system is determined by the performance of all components, especially limited by the weakest component. In the parallel model, the components of the system work in parallel, and the overall performance is a superposition of each component. From these two fundamental models, more complex models can be derived, such as the effective medium approximation methods, including the Maxwell-MG model [42] and the Bruggeman model [43]. The property functional equivalence for the series calculation model satisfies Equation (13), while that for the parallel calculation model satisfies Equation (14).

(13) $$\varphi_{total}=\frac{1}{\left(\sum_{i=1}^{N}\frac{V_{i}}{\varphi_{i}}\right)}$$

(14) $$\varphi_{total}=\sum_{i=1}^{N}V_{i}\varphi_{i}$$

where $\varphi_{total}$ represents the overall property functional of the system; $V_{i}$ represents the volume fraction of component *i* in a three-dimensional system, the area fraction in a two-dimensional system, or the length fraction in a one-dimensional system; $\varphi_{i}$ is the property functional of component *i*.

In the heat transfer system of FRP composite materials, the calculation of the system’s thermal conductivity needs particular attention, for a reasonable account of the contributions of fibers and resin to the equivalent thermal conductivity. Generally, the parallel model is suitable for calculating the system thermal conductivity of continuous fiber-reinforced composites aligned parallel to the fiber direction, while it could lead to significant overestimations for other types of composites. The series model is more appropriate for systems where fibers are evenly dispersed within the resin matrix [39]. Therefore, for the circular cross-section system of GFRPP reinforcement, the series model is applicable for the calculating thermal conductivity.

### 2.4. Numerical Solution Method

**Figure 3:**
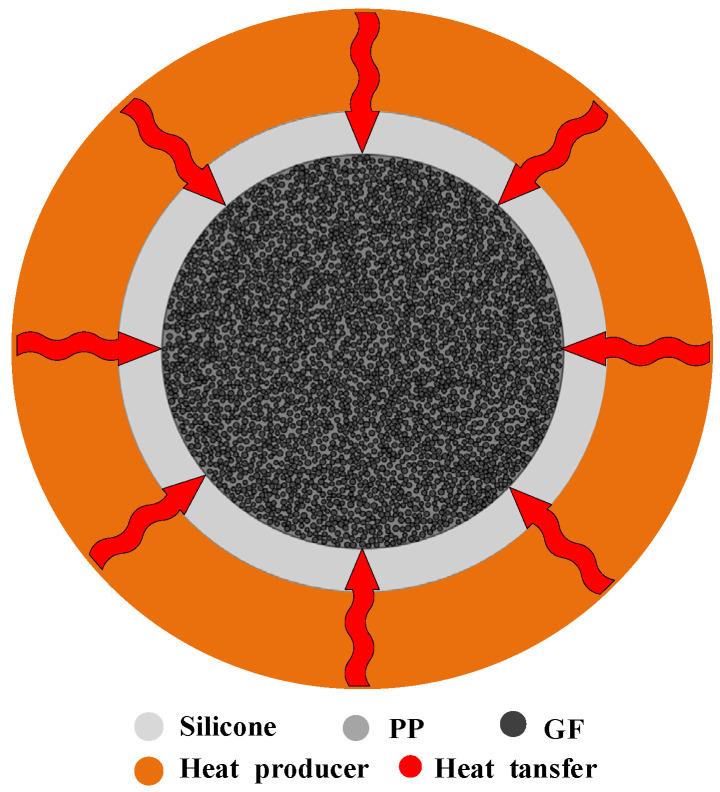
Silicone tube-GFRPP reinforcement two-dimensional circular heat system.

During the heating and bent processing, the flow of softening resin or significant cross-sectional shape changes of GFRPP reinforcement may occur, and a silicone tube with a wall thickness of 1.5 mm is generally utilized as a protective cover. Thus, the GFRPP reinforcement circular plane heating system (Figure 2) transitions to the silicone tube-GFRPP reinforcement circular plane heating system, as illustrated in Figure 3. Since the silicone tube is independent of the GFRPP reinforcement, the equivalence of their property functionals becomes more complex.

**Figure 4:**
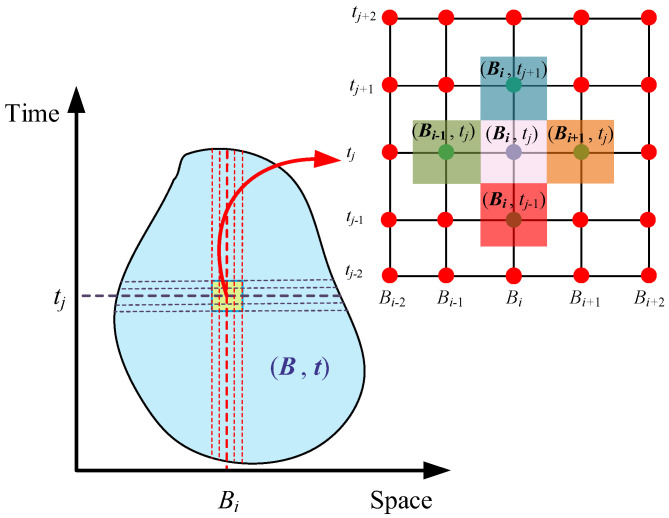
Discretization of continuous spatiotemporal systems.

When the homogeneity of the system is difficult to ensure, finding an analytical solution to a partial differential equation becomes challenging, and in some cases, an analytical solution may even be unattainable. A common approach to handling such partial differential equations is to approximate the solution using a numerical method. For the heat conduction equation, a frequently used numerical method is the finite difference method. The fundamental idea is to discretize the continuous spacetime system $\left(B,t\right)$, as illustrated in Figure 4. The continuous spacetime is discretized into numerous non-overlapping small spacetime intervals $\left(B_{i},t_{j}\right)\;\left(i,j\in Z^{+}\right)$. The relationship between the small spacetime intervals and the continuous spacetime is expressed in Equation (15). Homogeneity is exhibited by the points within $\left(B_{i},t_{j}\right)$, and their properties are uniquely determined by the center point of that spacetime interval, denoted as (*B_i_*, *t_j_*), which represents the coordinates of the mesh points used for the discretization of $\left(B,t\right)$.

(15) $$\left(B,t\right)=\overset{M}{\underset{j=1}{⋃}}\overset{N}{\underset{i=1}{⋃}}\left(B_{i},\;t_{j}\right),\left(M,N\in Z^{+}\right)$$

The derivative terms of the differential equation can be converted into algebraic relationships between grid points in the discretized system. The grid is typically divided into equal intervals, with the size of each interval referred to as the step size. For convenience, the step size in both directions of the two-dimensional plane is generally set to be equal and denoted as *h*. Similarly, time is divided into equal intervals, with the time step denoted as Δ*t*. Consequently, the coordinates in Figure 4 satisfy Equations (16) and (17).

(16) $$h=B_{i+1,\;x}-B_{i,x}=B_{i+1,y}-B_{i,y}$$

(17) $$∆t=t_{j+1}-t_{j}$$

where $B_{i,x}$ represents the *x*—component of the spatial coordinates of the center point (*B_i_*, *t_j_*), while $B_{i,y}$ denotes the *y*—component of the center point (*B_i_*, *t_j_*). Additionally, $t_{j}$ refers to the time coordinate of the center point (*B_i_*, *t_j_*).

The forward difference method is an explicit numerical technique commonly used to approximate solutions for partial differential equations with known boundary and initial conditions [43,44]. From the right side of the two-dimensional partial differential Equation (4), it can be observed that the temperature *T* is subjected to a second-order derivative for the spatial component. By applying the three-point central difference formula for the second-order derivative, Equations (18) and (19) can be derived. Additionally, from the left side of Equation (4), it is evident that the temperature *T* is first-order differentiated for time *t*. Using the two-point forward difference formula, Equation (20) can be obtained.

(18) $$\frac{\partial^{2}T}{\partial x^{2}}\approx \frac{T\left(x-h,y,t\right)-2T\left(x,y,t\right)+T\left(x+h,y,t\right)}{h^{2}}$$

(19) $$\frac{\partial^{2}T}{\partial y^{2}}\approx \frac{T\left(x,y-h,t\right)-2T\left(x,y,t\right)+T\left(x,y+h,t\right)}{h^{2}}$$

(20) $$\frac{\partial T}{\partial t}\approx \frac{T\left(x,y,t+∆t\right)-T\left(x,y,t\right)}{∆t}$$

Submitting Equations (18)~(20) into Equation (4), Equation (21) can be obtained.

(21) $$T\left(x,y,t+∆t\right)=T\left(x,y,t\right)+\frac{k∆t}{c\rho }\frac{T\left(x-h,y,t\right)+T\left(x+h,y,t\right)-4T\left(x,y,t\right)+T\left(x,y-h,t\right)+T\left(x,y+h,t\right)}{h^{2}}$$

The numerical method for iterative calculations based on Equation (21) is known as the forward difference method. In this equation, the parameters *k*, *c*, and *ρ* are independent of time, meaning they do not depend on the time component $t_{j}$ at $\left(B_{i},t_{j}\right)$. However, their values are dependent on the spatial location $B_{i}$ of the function being solved. Since the center point (*B_i_*, *t_j_*) characterizes the properties of the entire small spatial region, the property functional is uniquely determined by the spatial coordinates *B_i_* of the center point (*B_i_*, *t_j_*). The number of spatial coordinate components depends on the dimensionality of the space. In the two-dimensional space, the components ${(B}_{i,x},B_{i,y})$ determine the property functional within the spatial domain defined by $\left(B_{i},t_{j}\right)$.

## 3. Experiment Investigation

### 3.1. Materials and Test Apparatus

**Figure 5:**
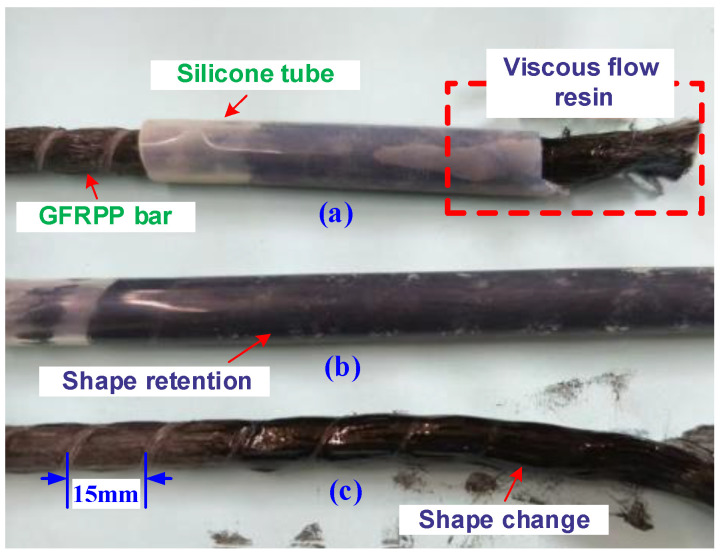
GFRPP rib heating softening: (**a**) viscous flow state; (**b**) shape retention; (**c**) shape change.

The GFRPP reinforcement used in the heating-softening test has a diameter of 9.8 mm, composed of modified PP resin and GFs with a diameter of approximately 20 µm, processed by melt pultrusion from GFRPP prepreg. The GFRPP reinforcements were developed by the Harbin Institute of Technology and produced by the Harbin Institute of Composite Materials, with a glass fiber volume fraction of 52%. The softening of the GFRPP reinforcement in the heated area manifests as a viscous flow state, resulting in a loss of rigidity, with significant cross-sectional shape changes under relatively small external forces, as depicted in Figure 5. To maintain the cross-sectional shape, a commercial silicone tube with a wall thickness of 1.5 mm is used to encase the outer perimeter of the heated area. The thermodynamic parameters of the experimental materials at room temperature are shown in Table 1.

**Figure 6:**
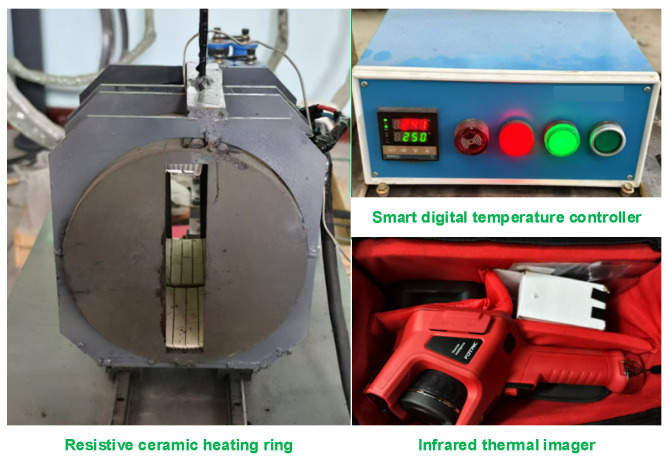
Heating and temperature field testing device.

The GFRPP reinforcement heating-softening test apparatus, as shown in Figure 6, consists of two main components, including a heating device and a temperature field measurement system. The heating device includes a resistive ceramic heating ring with a rated power of 4.5 kW and a smart digital temperature controller capable of handling power up to 6.5 kW. For temperature field measurement, a FOTRIC 348C+ handheld infrared thermal imager is used, which has an effective temperature measurement range of −20 °C to 650 °C.

### 3.2. Test Method and Results

**Figure 7:**
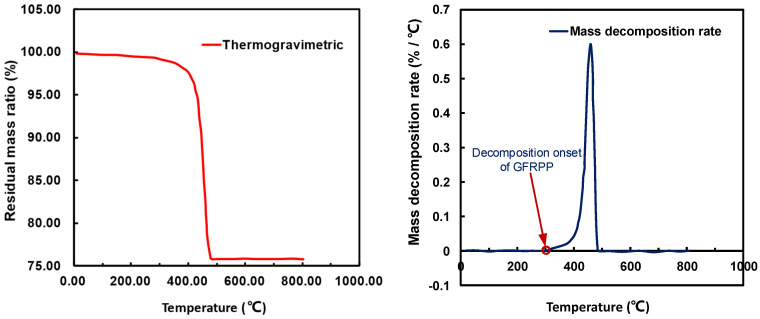
Thermogravimetric analysis of GFRPP [46].

According to the material composition of the GFRPP reinforcement, it is evident that the thermal softening of the reinforcement is primarily controlled by the PP resin, which has a softening temperature in the range of approximately 160 °C to 170 °C [45,46]. Thermogravimetric analysis (TGA) of the GFRPP reinforcement (Figure 7) indicates that the rate of weight loss begins to increase at around 300 °C [46], suggesting that the PP resin starts to decompose. To avoid property changes of the GFRPP reinforcement due to PP resin decomposition, it is necessary to keep the heating temperature below this threshold. Considering these factors, a heating temperature range of 190 °C to 300 °C is deemed appropriate.

**Figure 8:**
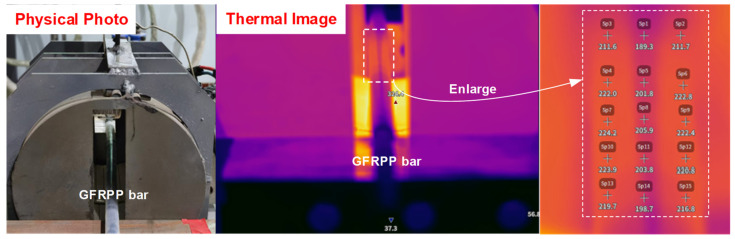
Temperature field contour measured by the infrared thermal imager at the midsection of the GFRPP reinforcement at a temperature of 190 °C indicated by the temperature control instrument.

**Figure 9:**
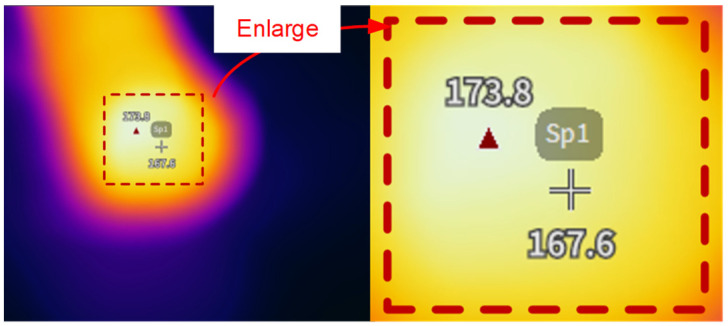
Temperature of the cross-sectional center of the GFRPP reinforcement at a temperature of 190 °C indicated by the temperature control instrument.

The heating temperature of the GFRPP reinforcement is controlled by a smart digital temperature control instrument, with ten sets of temperature control levels, including 190 °C, 200 °C, 210 °C, 220 °C, 230 °C, 240 °C, 250 °C, 260 °C, 270 °C, and 280 °C. However, actual temperature measurement reveals that due to the limitation of the temperature control probe arrangement, the measured heating temperature of the GFRPP reinforcement is higher than the temperature indicated by the digital temperature control instrument (Figure 8). To address the discrepancy between the measured temperature and the indicated temperature *T*_1_, an infrared thermal imager is employed to accurately measure the surface temperature field of the GFRPP reinforcement. As shown in Figure 9, 15 measurement points are evenly selected on the surface of the test GFRPP reinforcement, and the average temperature of these points is used as the actual measured temperature of the GFRPP reinforcement. This established a relationship between the measured temperature and the indicated temperature from the control instrument, allowing for the recalibration of the surface heating temperature of the GFRPP reinforcement. During the heating process, the following points need to be additionally clarified: (1) no external loads were applied to the reinforcement during heating; (2) due to the airtightness of the heating device and the dry internal environment, the influence of humidity inside the device can be considered negligible.

The cross-sectional center temperature of the GFRPP reinforcement serves as an indicator for determining whether it has fully softened. When the center temperature reaches the softening temperature of the PP resin, the GFRPP reinforcement is fully softened. The cross-sectional center temperature is measured using a thermal imager after cutting the GFRPP reinforcement (Figure 9). Due to the rapid cooling of the reinforcement surface, it is crucial to quickly capture and measure the temperature at the cutting location. For each temperature control level during the experiment, three valid data points are collected. The surface heating temperature of the GFRPP reinforcement *T*_r1_, the initial temperature *T*_0_, the center temperature *T*_center_, and the heating time *t*_test_ are presented in Table 2.

**Figure 10:**
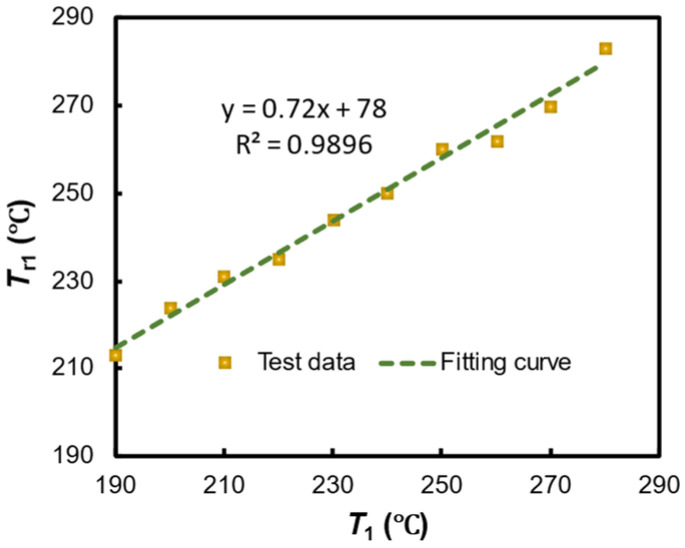
GFRPP bar heating temperature calibration.

From the data in Table 2, the relationship between the actual surface heating temperature of GFRPP reinforcement and the smart temperature control instrument indicated temperature can be calibrated, as illustrated in Figure 10 and represented by Equation (22). It is important to note that the calibration of the heating temperature for the GFRPP reinforcement provides accurate heating temperatures for both experimental and theoretical research.

(22) $$T_{r1}=0.72T_{1}+78$$

## 4. Numerical Modeling and Analysis

### 4.1. Establishment of the Numerical Model

The heating-softening model for the GFRPP reinforcement with a silicone tube cover should satisfy the following basic assumptions:

(1) The heat source effectively envelops the GFRPP reinforcement surface, with no temperature gradient along the longitudinal length direction.

(2) Heat transfer does not consider the thermal resistance effects at the interfaces of material components.

(3) The fibers in GFRPP reinforcement are continuous in the longitudinal direction, with a neglectable fiber deflection angle.

(4) The cross-section of the GFRPP reinforcement and GFs is circular, and the circular silicone tube has a uniform wall thickness of 1.5 mm.

(5) GFs are randomly and uniformly dispersed within the PP resin.

(6) There are neglectable inner defects such as internal voids within the GFRPP reinforcement.

**Figure 11:**
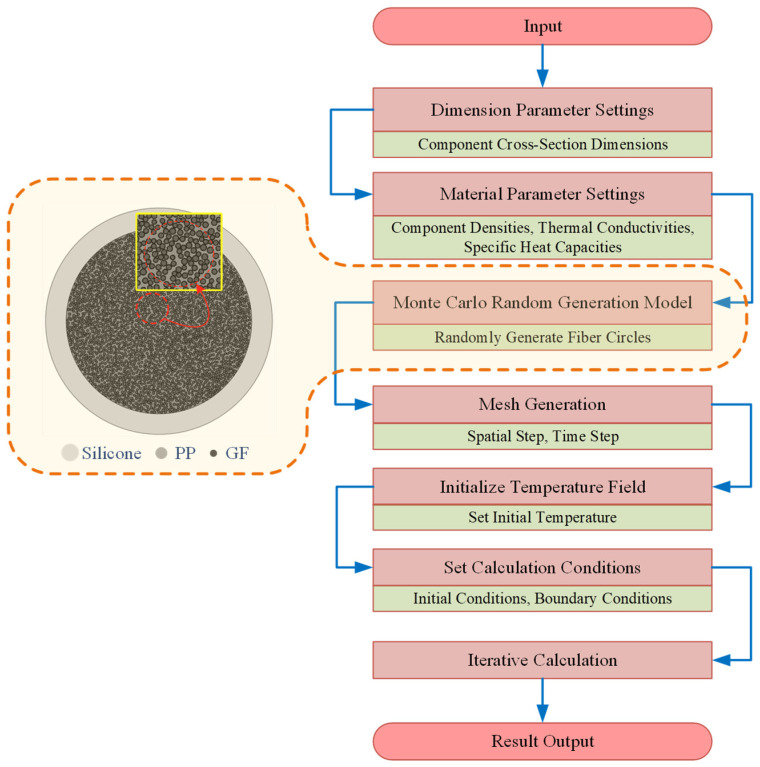
Modeling and calculation flowchart.

Based on the above assumptions, the heating-softening model for the GFRPP reinforcement with a silicone tube cover can be treated as a two-dimensional circular plane model, as shown in Figure 3. Using the Monte Carlo Method, circular fibers with a non-overlapping area ratio of 52% can be randomly and uniformly generated within the circular GFRPP reinforcement cross-section. The measured diameter of the glass fiber is approximately 20 μm. However, numerical modeling has shown that generating fibers with their actual size incurs high computational time costs. Fortunately, it is validated that scaling up the fiber diameters has a minimal impact on the calculation results, as indicated in Table 3. By controlling the diameter of the glass fiber to 100 μm in the numerical model, a good balance between computational efficiency and accuracy can be achieved. In the circular GFRPP cross-section, the other area that is not occupied by the circular fibers represents the PP resin. The silicone tube cover is the region between the circular GFRPP and the surrounding circle. A schematic diagram of the cross-sectional model of the heating system and the calculation process is shown in Figure 11.

### 4.2. Numerical Model Calculation

The temperature field calculations for the heating system are conducted using MATLAB R2020b, employing a forward difference method for iterative computation. The heating temperatures for the model are taken from the measured values in Table 2, with the initial condition set to the room temperature *T*_0_. The iterative computation is terminated when the central temperature *T*_center_ of the model reaches the measured central temperature specified in Table 2.

The forward difference method approximates derivatives through algebraic relationships at discrete points, leading to truncation errors. These errors depend on the values of the spatial step size *h* and the time step size Δ*t*, which influence the convergence of the solution. According to von Neumann stability analysis [47], when *h* and Δ*t* satisfy the inequality in Equation (23), the truncation error of the forward difference expression in Equation (21) can be effectively controlled, thereby ensuring the computational stability of the numerical solution.

(23) $$\frac{D∆t}{h^{2}}<0.5$$

In Equation (23), *D* represents the thermal diffusivity, calculated according to Equation (24).

(24) $$D=\frac{k}{\rho c}$$

where *k* is the material thermal conductivity, with a unit of W/(m·K); *c* represents the specific heat capacity of materials, with a unit of kJ/(kg·K); *ρ* is the material density, with a unit of kg/m^3^.

Based on the relevant thermodynamic parameters from Table 1, the spatial step size *h* is set to 0.1 mm, and the time step size Δ*t* is set to 0.005s. This choice ensures that the model exhibits good convergence and efficient computational performance. The softening time *t*_m_ obtained from the numerical model calculations is shown in Table 4.

### 4.3. Modification of the Thermal Diffusivity

**Figure 12:**
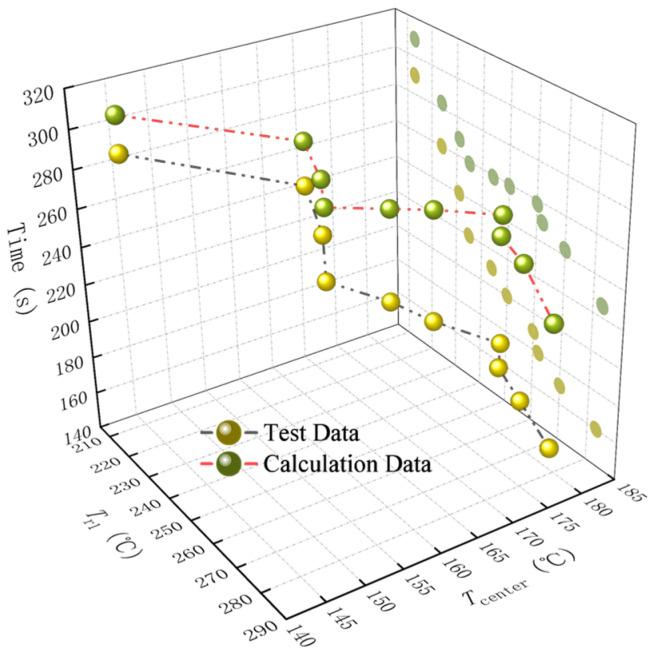
System GFRPP reinforcement softening time test and calculated value comparison.

According to the experimental and numerical results in Table 2 and Table 4, their comparison of the heating-softening time results is shown in Figure 12, which indicates that the calculated softening time is higher than the experimental value and the difference becomes more pronounced at higher temperatures. In the numerical calculations, the thermal diffusivity coefficient *D* is treated as a steady-state parameter. However, the thermal diffusivity of materials differs between room temperature and elevated temperature conditions, and changes in material morphology induced by elevated temperatures can lead to significant variations in thermal diffusivity [48]. Studies have shown that the thermal diffusivity of polymers can increase by 300% to 400% at temperatures over the melting temperature, due to the rearrangement of molecular chains [49].

In the GFRPP-silicone tube system, the GFs and silicone tube experience minimal morphological changes during the heating process, and thus their thermal diffusivity changes are limited. Existing research has indicated that the thermal conductivity of silicone remains relatively unchanged at high-temperature conditions [50]. However, the softening of PP resin leads to alterations in the arrangement of molecular chains, which increases its thermal diffusivity. Additionally, GFRPP exhibits enhanced flow properties after melting, making the system in a flow-solid coupling state, which goes beyond the typical scope of solid heat transfer. Therefore, it is essential to modify the thermal diffusivity of the GFRPP reinforcement. The thermal diffusivity of the GFRPP reinforcement is primarily determined by the thermal diffusivities of PP resin and GFs, and the equivalent calculation model can be approximately seen as a series calculation model. When modifying the thermal diffusivity of GFRPP reinforcement, it is possible to adjust the thermal diffusivities of both GFs and PP simultaneously or to focus on correcting just one of them. Both approaches can yield similar results, but the latter is simpler and easier to implement.

Existing research has indicated that modifying the thermal diffusivity coefficient of GFs has significant advantages: (1) Since the thermal diffusivity coefficient of GFs is lower, as indicated by Equation (13), even slight changes can lead to considerable variations in GFRPP’s thermal diffusivity. This means that adjusting the GF thermal diffusivity coefficient can effectively reflect the thermal behavior of GFRPP. (2) Overly large adjustments to the thermal diffusivity coefficient of PP resin may lead to convergence issues in numerical calculations. In such cases, it becomes necessary to adjust the analysis step size and time step size based on the modified values, significantly altering the computational structure of the analysis model. In contrast, small adjustments to the thermal diffusivity coefficient of GFs can achieve similar results, and are less likely to cause convergence problems, eliminating the need for adjusting the computational structure of the analysis model.

Therefore, the thermal diffusivity coefficients of the silicone tube and PP resin are kept unchanged, while only that of GFs is adjusted. Given the heating temperature *T*_r1_, initial temperature *T*_0_, center temperature *T*_center_, and heating time *t* = *s*, based on Equations (12) and (24), the thermal diffusivity coefficient of GFRPP can be expressed as Equation (25), which describes the relationship between the thermal diffusivity coefficient *D* at time *t* = *s* and a relative temperature difference *A*.

(25) $$D=F\left(A,s\right)\;\;\;\;\;\;A=\frac{T_{r1}-T_{center}}{T_{r1}-T_{0}}$$

However, solving the specific functional relationship in Equation (25) is relatively complex. In the forward difference method, the thermal diffusivity coefficient *D* can be replaced by the mean thermal diffusivity coefficient $\bar{D}$ over the entire heating period. According to Equation (26), when *D* is a steady-state parameter, the mean $\bar{D}$ is equal to *D*.

(26) $$\bar{D}=\frac{\int_{0}^{t_{test}}Dds}{t_{test}}$$

**Figure 13:**
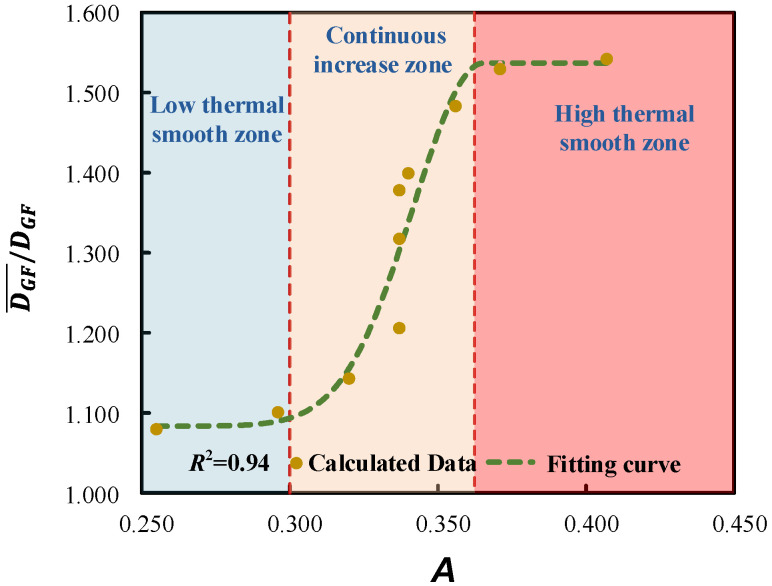
The relationship between $\frac{\bar{D_{GF}}}{D_{GF}}$ and *A*.

This means that the thermal diffusivity coefficient of glass fibers $\bar{D_{GF}}$ for GFRPP over the entire heating period can be determined using the bisection method, ensuring that the calculation time aligns with the experimental time. The specific calculation results are shown in Table 5. The relationship between the dimensionless mean thermal diffusivity coefficient $\bar{D_{GF}}$/$D_{GF}$ and the relative temperature difference *A* is illustrated in Figure 13, which reflects the overall trend of the thermal conduction in the GFRPP system as it varies with temperature difference.

As can be seen from Figure 13, the changes in the thermal diffusivity exhibit significant nonlinear features, which can be divided into three major regions, including the low-thermal-diffusivity-gently varying region, the continuous-thermal-diffusivity-increase region, and the high-thermal-diffusivity-gently varying region.

(1) The low-thermal-diffusivity-gently varying region. When *A* ≤ 0.3, the thermal diffusivity of the GFRPP system does not differ significantly from that at room temperature. In this case, the thermal diffusivity at room temperature can be directly used for calculations.

(2) The continuous-thermal-diffusivity-increase region. When 0.3 < *A* ≤ 0.366, the increase in thermal diffusivity first accelerates with rising heating temperature and then decelerates. This characteristic is primarily influenced by the softening degree of PP resin at different temperatures. In the early stage of heating, the softening state of PP resin molecular chains changes significantly with elevated temperatures, leading to an accelerated rate of increase in thermal diffusivity. In the later stage of heating, PP resin gradually approaches a fluid state, and the molecular chain structure stabilizes, resulting in a decelerated rate of increase in thermal diffusivity.

(3) The high-thermal-diffusivity-gently varying region. When *A* > 0.366, the resin is fully softened, and the molecular chain structure maintains a stable form, leading to a steady-state thermal diffusivity.

Based on the data points in Table 5 and Figure 13, the modified average thermal diffusivity of GFs throughout the entire heating period can be fitted, as expressed in Equation (27), where when *A* ≤ 0.366, *B* is set to 0.023, and when *A* > 0.366, B→∞.

(27) $$\bar{D_{GF}}=D_{GF}\left(1.084+0.454exp\left[-\frac{{\left(A-0.366\right)}^{2}}{2B^{2}}\right]\right)$$

**Figure 14:**
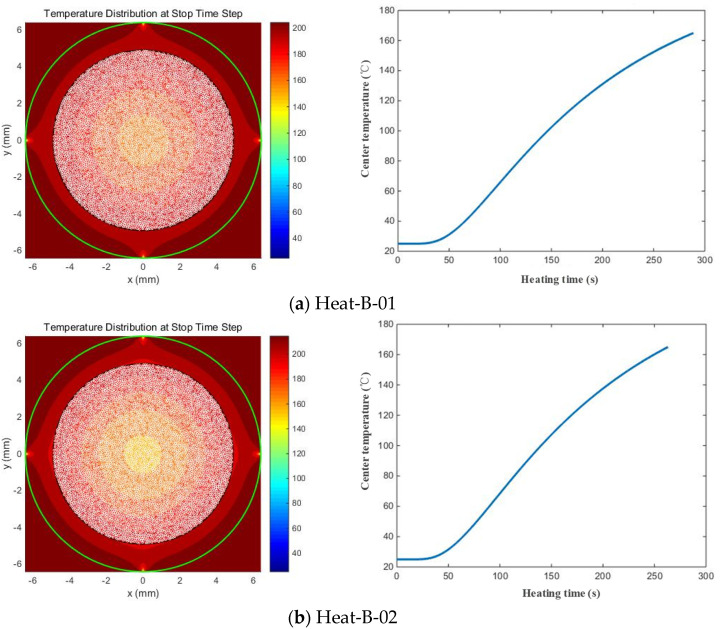

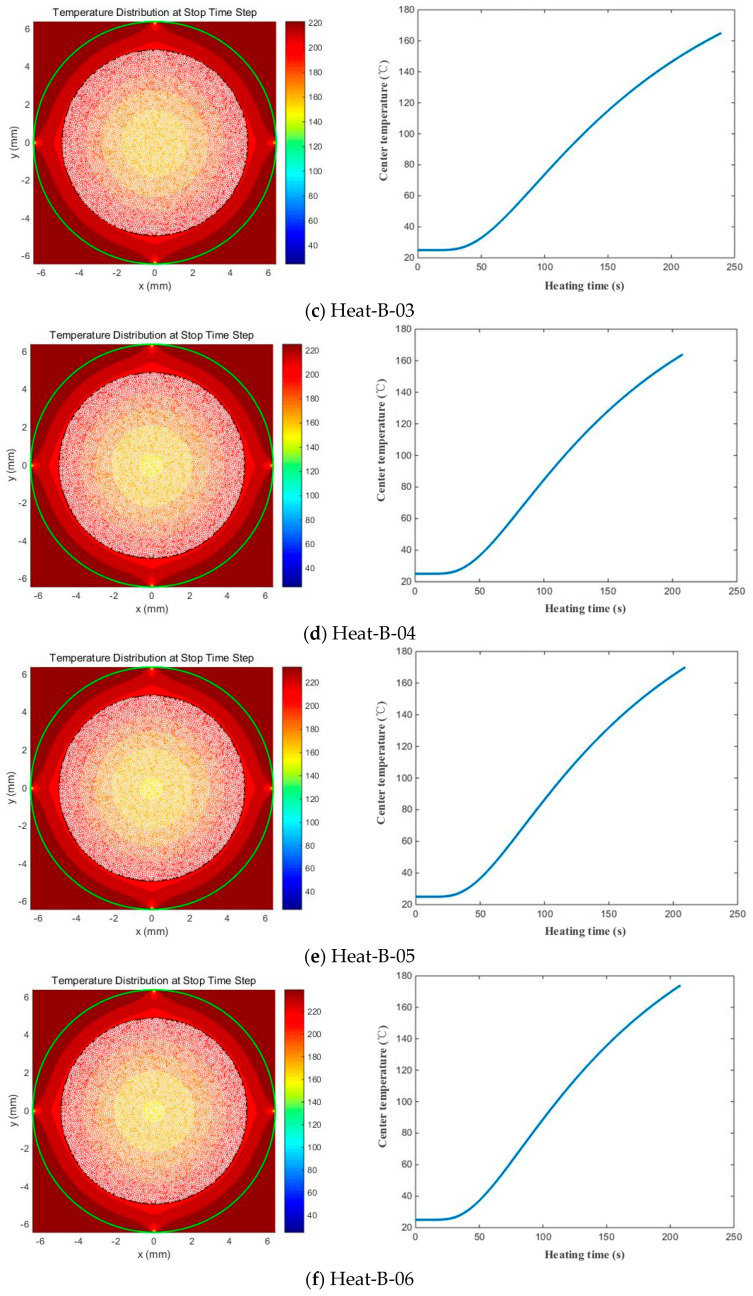

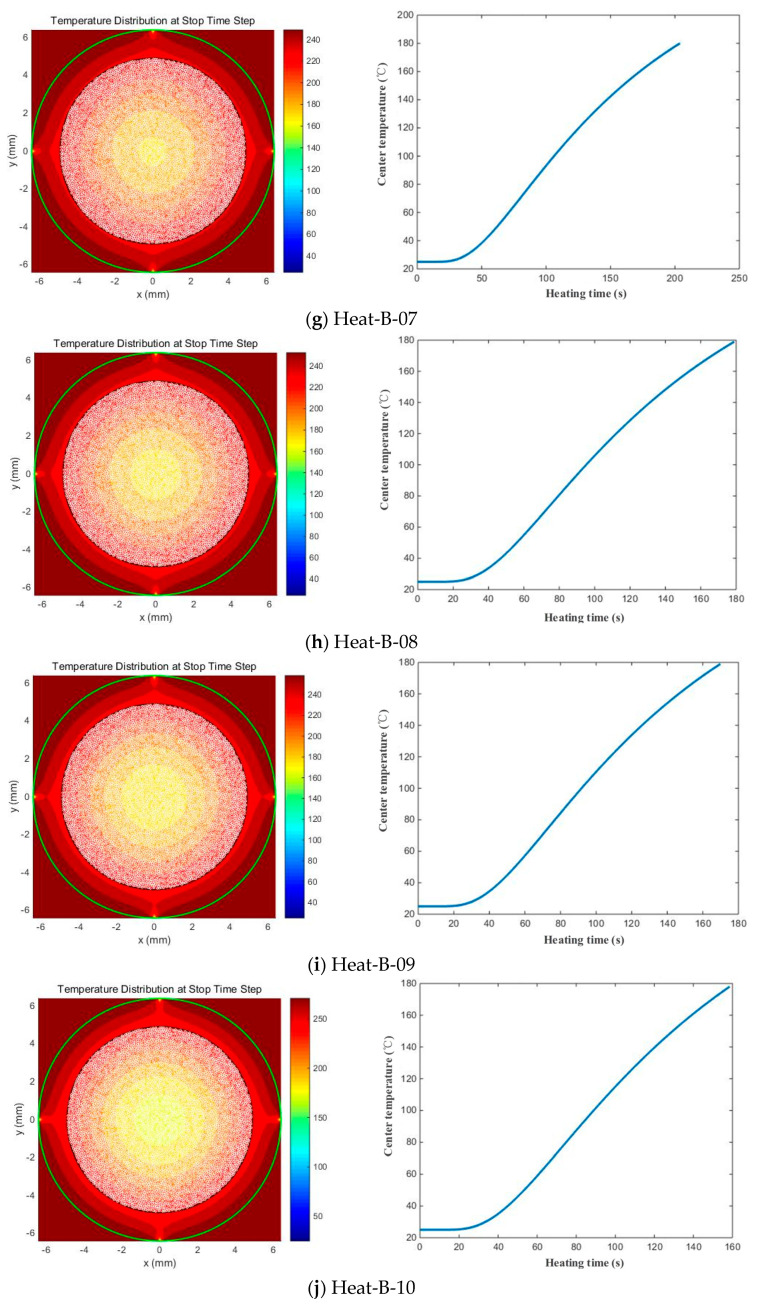
Temperature field distribution contour and the relationship between GFRPP center temperature and heating time by the numerical model with modified GF thermal diffusivity.

By substituting the modified GF thermal diffusivity from Equation (27) into the numerical model, the numerical temperature field distribution contours for each model can be obtained, as well as the relationship between the GFRPP reinforcement center temperature and heating time, which are depicted in Figure 14.

During the heating process, the GFRPP reinforcement center temperature initially remains constant for a period, whose duration period is not significantly changed with the increase in heating temperature. This is because heat transfer is not instantaneous but occurs along specific paths, saying that the increase rate in center temperature depends not only on the heating temperature but also on the length of the heat transfer path and the comprising material composition. According to the series calculation model, the thermal conductivity rate of the system is mainly influenced by the components with lower thermal conductivity in the path. GFs with relatively low thermal conductivity significantly delay the heat transfer within the GFRPP reinforcement, resulting in a temperature difference between the center and the surface. After equivalent modification of the GF thermal diffusivity, the comparative validation of the experimental and numerical heating-softening time for GFRPP reinforcement is shown in Table 6, indicating a high consistency and demonstrating the validity of the equivalent GF thermal diffusivity modification approach in the numerical model.

### 4.4. Parameter Analysis

**Figure 15:**
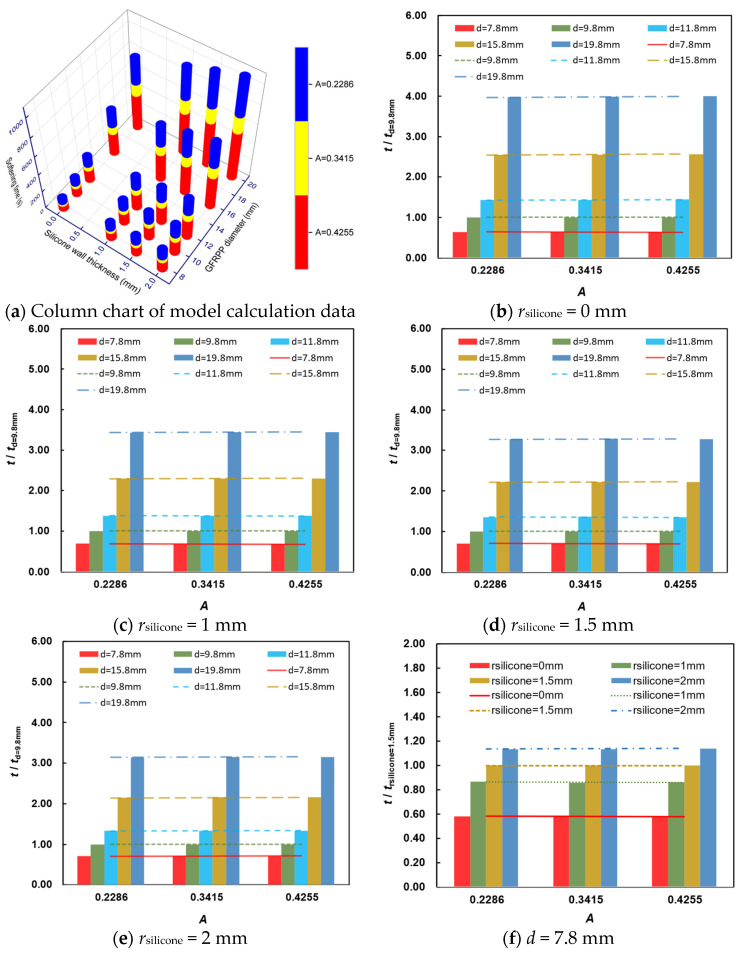

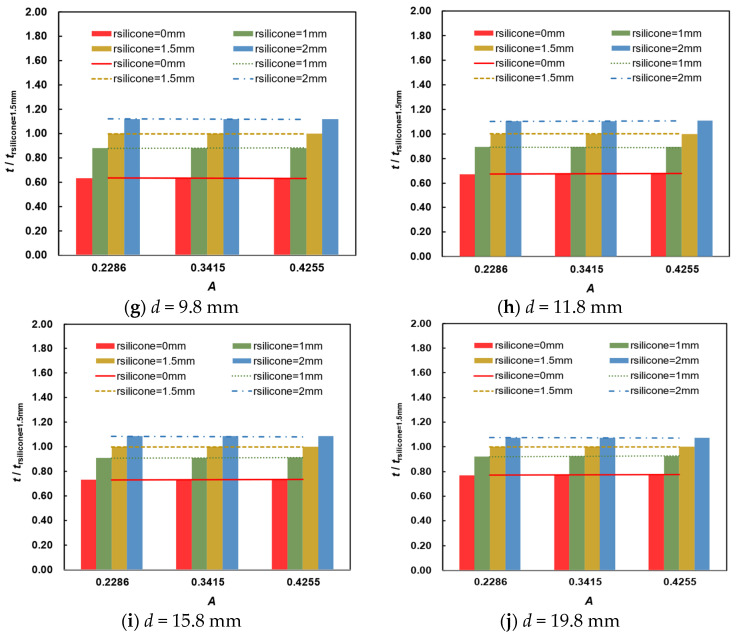
Influences of GFRPP diameter and silicone tube wall thickness on the softening time.

The condition for complete softening of GFRPP reinforcement is that the center temperature reaches 160 °C. Factors affecting the softening time of GFRPP reinforcement include the heating temperature, GFRPP initial temperature, GFRPP fiber volume content, GF diameter, GFRPP reinforcement diameter, and silicone tube wall thickness, among which the effects of GF diameter, heating temperature, and GFRPP initial temperature have been detailed in Section 4.1 and Section 4.3. GFs act as a filler in the polymer matrix, whose volume content, size, and shape may significantly affect the thermal conductivity of polymer composites. Generally, a high content of filler can suppress the crystallization of the polymer matrix, thereby reducing the thermal conductivity of the composite materials [51]. Therefore, GFRPP reinforcement with higher fiber content exhibits lower thermal conductivity. Regarding the influence of filler size, significant effects can be observed when it reaches the nanoscale [39]. Since the fiber size far exceeds the nanoscale, the effect of fiber size variation on the thermal conductivity can be considered negligible, which is also validated in Section 4.1. Additionally, the fiber volume content of GFRPP reinforcement is an optimal solution, considering the preparation process, mechanical performance requirements, and production costs, and it generally varies slightly. Thus, the major parameters that need to be investigated are the GFRPP reinforcement diameter and the silicone tube wall thickness. To investigate their effects, the GFRPP diameter *d* is selected at five levels, including 7.8 mm, 9.8 mm, 11.8 mm, 15.8 mm, and 19.8 mm, and the silicone tube wall thickness *r*_silicone_ is selected at four levels, including 0 mm, 1 mm, 1.5 mm, and 2 mm. Serious numerical calculations of the GFRPP reinforcement heating-softening time *t*_m_ are conducted, and their details are presented in Table 7. Additionally, the influences of GFRPP diameter and silicone tube wall thickness on the softening time are also comparatively analyzed, as illustrated in Figure 15.

As shown in Figure 15a, with the increase in GFRPP reinforcement diameter and silicone tube wall thickness, the heating-softening time increases. In contrast, an increase in the relative temperature difference *A* can accelerate the heating-softening process. According to Figure 15b–e, the relative heating-softening time of GFRPP reinforcement with different diameters varies proportionally with changes in the relative temperature difference *A*, which indicates that the effect of GFRPP reinforcement diameter *d* on the heating-softening time is independent of temperature difference *A*. From Figure 15f–j, the relative heating-softening time of GFRPP reinforcement with different silicone tube wall thickness also exhibits a proportional and synchronous variation with the relative temperature difference *A*, which also indicates an effect of the silicone tube wall thickness independent of temperature difference *A*.

**Figure 16:**
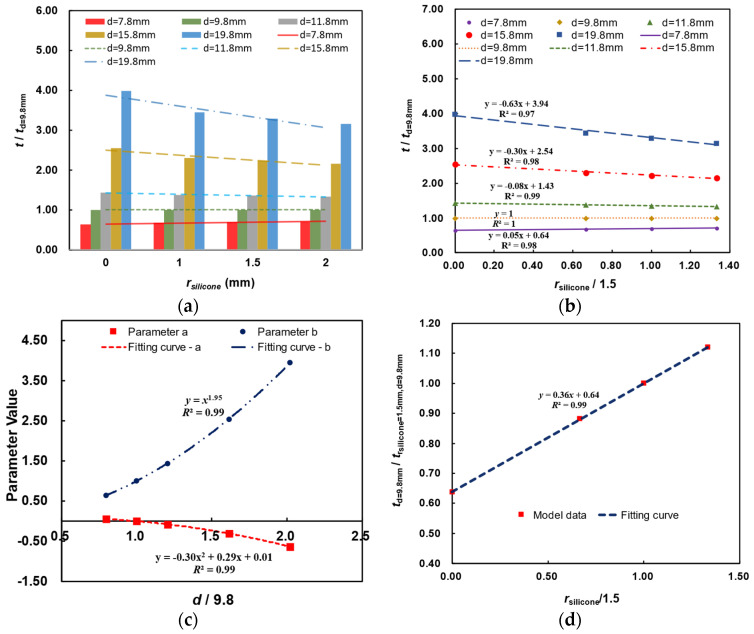
Correlative influences of GFRPP reinforcement diameter and silicone tube wall thickness on heating-softening time: (**a**) column chart of softening time (*A* = 0.3415); (**b**) vibration law of softening time (*A* = 0.3415); (**c**) relationship of values *a*, *b*, and the relative GFRPP reinforcement diameter; (**d**) relationship between the heating-softening time and silicone tube wall thickness at a GFRPP reinforcement diameter of 9.8 mm.

Due to the independent influence of GFRPP reinforcement diameter *d* and silicone tube wall thickness *r*_silicone_ with temperature difference *A*, taking *A* = 0.3415 as an example, the correlation of the influences of *d* and *r*_silicone_ on the heating-softening time is analyzed, as illustrated in Figure 16a,b. As can be seen from Figure 16b, with the increase in GFRPP reinforcement diameter, the correlative influences between *d* and *r*_silicone_ on the relative heating-softening time gradually become more significant. Thus, the relative heating-softening time of the GFRPP reinforcement-silicone tube system considering the correlation between *d* and *r*_silicone_ can be established by data fitting in Figure 16b, as expressed in Equation (28).

(28) $$\frac{t}{t_{d=9.8}}=a\left(\frac{r_{silicone}}{1.5}\right)+b$$

where *a* and *b* are dimensionless coefficients related to the relative GFRPP reinforcement diameter *d*/9.8 and the relative silicone tube wall thickness *r*_silicone_/1.5. From the functions of fitting lines in Figure 16b for different compositions of *d* and *r*_silicone_, the values of *a* and *b* are shown in Table 8.

Further, using the data in Table 8, the relationships between values of *a*, *b*, and the relative GFRPP reinforcement diameter *d*/9.8 are depicted in Figure 16c, which are also fitted in the figure. Substituting the fitting formula of values of *a* and *b* into Equation (28) can yield Equation (29), which presents the heating-softening time prediction for the GFRPP-silicone system with different reinforcement diameters and tube wall thicknesses.

(29) $$\frac{t}{t_{d=9.8}}=\left[-0.30{\left(\frac{d}{9.8}\right)}^{2}+0.29\left(\frac{d}{9.8}\right)+0.01\right]\frac{r_{silicone}}{1.5}+{\left(\frac{d}{9.8}\right)}^{1.95}$$

It should be noted that Equation (29) mainly focuses on the influence of GFRPP reinforcement diameters on the heating-softening time, based on different specific silicone tube wall thicknesses, where a singular point exists when *d* = 9.8 mm, saying that the influence of silicone tube wall thickness variation cannot be reflected because the coefficient *a* in Equations (28) and (29) keeps constant as zero. In this case, the influence of silicone tube wall thickness variation needs to be specifically investigated by taking the numerical models MH25, MH26, MH27, and MH28 (with *A* = 0.3415, *d* = 9.8 mm, and varying *r*_silicone_) in Table 7 for analyzing the influence of silicone tube wall thickness, as depicted in Figure 16d. The increase in the thickness of the silicone tube effectively extends the distance of heat conduction, thereby increasing the heating time. The relationship between the heating-softening time and silicone tube wall thickness at a GFRPP reinforcement diameter of 9.8 mm can be obtained, as expressed in Equation (30).

(30) $$\frac{t_{d=9.8mm}}{t_{r_{silicon}=1.5mm,\;\;\;d=9.8mm}}=0.36\left(\frac{r_{silicone}}{1.5}\right)+0.64$$

## 5. Simplified Model for Calculating Heating Softening Time

### 5.1. Simplified Semi-Analytical and Semi-Empirical Calculation Model

The above analysis has indicated that it is not feasible to directly solve the analytical heating-softening time using Equation (12) in Section 2.3.2, due to complex parameter changes during the heating-softening process of the GFRPP reinforcement-silicone tube system, particularly the variations of the thermal diffusion coefficient at different elevated temperatures that is caused by the melting and molecular chain rearrangement of PP resin at high temperatures. Thus, a semi-analytical and semi-empirical calculation model is proposed by utilizing Equation (12) in conjunction with experimental and numerical analysis results.

**Figure 17:**
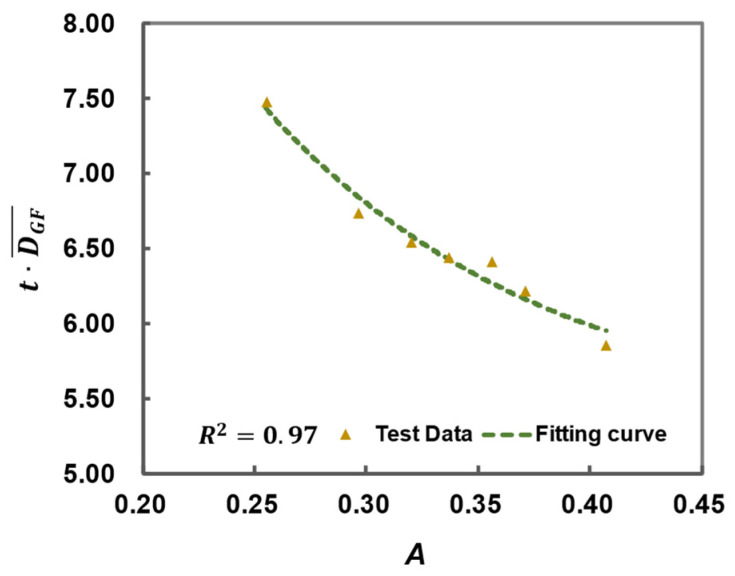
The relationship between $t\cdot \bar{D_{GF}}$ and *A*.

Taking the experimental GFRPP reinforcement diameter of 9.8 mm and silicone tube thickness of 1.5 mm, it can be derived from Equation (12) that the relationship between time *t* and relative temperature difference *A* satisfies Equation (31).

(31) $$t=\frac{Z\left(A\right)}{D_{all}}$$

where $D_{all}$ is the equivalent thermal diffusivity of the system and *Z* is a function of *A*. According to the numerical investigation in Section 4.3, it has been shown that the variation of $D_{all}$ is mainly positively influenced by GF thermal diffusivity $\bar{D_{GF}}$. Assuming that there is a functional relationship between $D_{all}$ and $\bar{D_{GF}}$, Equation (31) can be transformed into Equation (32).

(32) $$t\cdot \bar{D_{GF}}=W\left(A\right)$$

where *W*(*A*) is a modified function of *Z*(*A*) that involves the relationship between $D_{all}$ and $\bar{D_{GF}}$, which can be fitted using experimental data points in Table 2. As shown in Figure 17, based on the experimental data fitting, the heating-softening time of the GFRPP-silicone tube system with a reinforcement diameter of 9.8 mm and tube thickness of 1.5 mm is expressed in Equation (33).

(33) $$t_{r_{silicon}=1.5,d=9.8mm}=\frac{5.29+15.44exp\left(-7.85A\right)}{\bar{D_{GF}}}$$

where $\bar{D_{GF}}$ is the modified average thermal diffusivity of GFs, determined in Equation (27).

From Equations (27), (29), (30), and (33), the general formula for calculating the heating-softening time of a GFRPP reinforcement-silicone tube system can be expressed as Equation (34).

(34) $$t=\frac{\left[0.36\left(\frac{r_{silicone}}{1.5}\right)+0.64\right]\left[5.29+15.44exp\left(-7.85A\right)\right]\left\{\left[-0.30{\left(\frac{d}{9.8}\right)}^{2}+0.29\left(\frac{d}{9.8}\right)+0.01\right]\left(\frac{r_{silicone}}{1.5}\right)+{\left(\frac{d}{9.8}\right)}^{1.95}\right\}}{D_{GF\;}\left\{1.084+0.454exp\left[-\frac{{\left(A-0.366\right)}^{2}}{2B^{2}}\right]\right\}}$$

where *t* is the required heating-softening time, with a unit of s; *r*_silicone_ is the silicone tube wall thickness, with a unit of mm; *d* is the GFRPP reinforcement diameter, with a unit of mm; *A* is the temperature difference, taken as (*T*_r1_ − *T*_center_)/(*T*_r1_ − *T*_0_), *T*_r1_ is the heating temperature, *T*_0_ is the initial (room) temperature, *T*_center_ is the required softening center temperature as 160 °C; *B* is a coefficient for equivalent thermal diffusivity modification of GFs, taken as 0.023 when *A* ≤ 0.366 or as ∞ when *A* > 0.366; *D*_GF_ is the thermal diffusivity of the GFs, calculated by Equation (24) and the GF thermodynamic parameters in Table 1.

### 5.2. Validation of the Calculation Model

The numerical models in Table 7 are utilized for conducting a comparative validation of the simplified semi–analytical and semi-empirical calculation model. By substituting model parameters into Equation (34), the simplified calculation heating-softening time *t*_eq_ can be obtained, and the model parameters, simplified calculation parameters, numerical heating-softening time *t*_m_, and simplified calculation time *t*_eq_, are shown in Table 9, from which it can be seen that *t*_eq_ coincides well with *t*_m_, with an average ratio of 1.010, a standard deviation of 0.026, and a coefficient of variation of 0.026, indicating that the simplified calculation model has high accuracy. Therefore, Equation (34) can be used for fast calculation of the required heating-softening time of GFRPP reinforcement-silicone tube at different heating temperatures.

## 6. Conclusions

This paper investigated the thermal conductivity and heating-softening time of the GFRPP reinforcement-silicone tube system through experiment investigation and numerical simulation. Based on the experimental data, an equivalent modified thermal diffusivity coefficient of glass fiber was proposed to account for the time-temperature-dependent heat conductivity of GFRPP reinforcement. Based on the unsteady heat conduction equation, an analytical solution to the heat conduction equation for a homogeneous circular cross-section was derived. Further, by integrating experimental data and numerical simulations, a semi-analytical and semi-empirical theoretical model for calculating the heating-softening time of the GFRPP reinforcement-silicone tube system was also proposed. The main conclusions are as follows:

(1) The thermal diffusivity coefficient of GFRPP reinforcement exhibits nonlinear variation with the relative temperature differences due to PP resin softening at elevated temperatures. Compared with PP resin thermal diffusivity modification, equivalent thermal diffusivity modification of GFs can relatively accurately reflect the thermal diffusivity variation of GFRPP reinforcement, which avoids potential convergence problems and eliminates computational structure adjustments, such as analysis step size and time step size adjustments, of the numerical model, thus exhibiting high efficiency.

(2) The thermal diffusivity coefficient of the GFRPP reinforcement-silicone tube system is time- and heating-temperature-dependent, making it challenging to determine a functional relationship between the thermal diffusivity coefficient and heating temperature and time. Consequently, a heating-temperature-dependent average GFRPP thermal diffusivity coefficient over the entire heating period is proposed, reflecting the overall trend of the thermal conduction of GFRPP reinforcement and making the numerical analysis feasible and relatively accurate.

(3) The influences of GFRPP reinforcement diameter and silicone tube wall thickness on the heating-softening time are independent of the heating temperature, but they are correlative. Based on the experimental data and the numerical analysis results, a simplified semi-analytical and semi-empirical calculation model for predicting the heating-softening time of the GFRPP reinforcement-silicone tube system is proposed, and it is consistent with the simulation results, with an average deviation of 1.0% and a coefficient of variation of 0.026, demonstrating the model’s strong potential for engineering applications.

(4) Due to limitations in research conditions and experimental methods, it is currently not feasible to precisely measure and simulate the non-uniformity of fiber distribution and its specific impact on thermal conductivity. The assumption of uniform fiber distribution in this study is a simplified approach based on existing research foundations and technical feasibility, aimed at facilitating model establishment and calculations. In subsequent research, it will be essential to prioritize this issue and strive to improve experimental methods to more accurately reflect real-world conditions.

This work provides a simplified heating-softening time calculation model for fast prediction of the heating time for GFRPP reinforcement with various diameters, silicone tube covers, and heating temperatures, facilitating a fast processing optimization of heating-softening-bending of GFRPP reinforcement in engineering applications. However, while the heating-softening behavior of fiber-reinforced thermoplastic polymer composites is closely related to the thermal behavior of the comprising fibers and resin matrix, it should also be noted that the simplified heating-softening time calculation model for GFRPP reinforcement cannot be directly used for other polymer composites with different fibers or a different resin matrix, and appropriate revisions of the parameters in the calculation model are needed.

## Figures and Tables

**Table 1:** Thermodynamic parameters of materials at room temperature.

Materials	Density [g/cm^3^]	Specific Heat Capacity [J/(g·K)]	Thermal Conductivity [W/(m·K)]
PP	0.91	1.60	0.24
GF	2.50	0.84	0.05
Silicone	1.10	1.20	0.20

**Table 2:** Results of heating-softening experiments for GFRPP reinforcement.

Test Number	*T*_1_ [°C]	*T*_r1_ [°C]	*T*_0_ [°C]	*T*_center_ [°C]	*t*_test_ [s]
Heat-B-01	190	213	25	165	290
Heat-B-02	200	224	25	165	260
Heat-B-03	210	231	25	165	240
Heat-B-04	220	235	25	164	220
Heat-B-05	230	244	25	170	210
Heat-B-06	240	250	25	174	200
Heat-B-07	250	260	25	180	190
Heat-B-08	260	264	25	179	180
Heat-B-09	270	270	25	179	170
Heat-B-10	280	283	25	178	160

*T*_1_—temperature indicated by the temperature control instrument; *T*_r1_—measured surface heating temperature of the GFRPP reinforcement; *T*_0_—initial (room) temperature; *T*_center_—measured cross-sectional center temperature of the GFRPP reinforcement; *t*_test_—heating time.

**Table 3:** Model computational efficiency and accuracy.

Diameter of the Fibers in Numerical Model (μm)	Result Error (%)	Model Runtime (min)
20	0	11,858
50	2.5	307
100	4.0	21

The result error is the relative error between the softening times calculated by numerical models with different fiber diameters and those with a fiber diameter of 20 μm.

**Table 4:** Numerical calculation parameters and results.

Model Number	*T*_r1_ [°C]	*T*_0_ [°C]	*T*_center_ [°C]	*t*_m_ [s]
Heat-B-01	213	25	165	309
Heat-B-02	224	25	165	283
Heat-B-03	231	25	165	269
Heat-B-04	235	25	164	259
Heat-B-05	244	25	170	259
Heat-B-06	250	25	174	259
Heat-B-07	260	25	180	258
Heat-B-08	264	25	179	250
Heat-B-09	270	25	179	243
Heat-B-10	283	25	178	226

**Table 5:** Calculated parameters related to glass fiber for the test GFRPP reinforcements.

Test Number	A	$\bar{D_{GF}}$ [W·mm^2^]	$\frac{\bar{D_{GF}}}{D_{GF}}$
Heat-B-01	0.255	0.0257	1.080
Heat-B-02	0.296	0.0262	1.101
Heat-B-03	0.320	0.0272	1.143
Heat-B-04	0.338	0.0287	1.206
Heat-B-05	0.337	0.0313	1.317
Heat-B-06	0.338	0.0328	1.378
Heat-B-07	0.340	0.0333	1.399
Heat-B-08	0.356	0.0353	1.483
Heat-B-09	0.371	0.0364	1.529
Heat-B-10	0.407	0.0367	1.542

**Table 6:** Validation of the numerical model with modified GF thermal diffusivity.

Test Number	Experimental Time (s)	Calculated Time (s)	Calculated Time/Experimental Time
Heat-B-01	290	289	0.997
Heat-B-02	260	263	1.012
Heat-B-03	240	239	0.996
Heat-B-04	220	208	0.945
Heat-B-05	210	209	0.995
Heat-B-06	200	208	1.040
Heat-B-07	190	204	1.074
Heat-B-08	180	179	0.994
Heat-B-09	170	170	1.000
Heat-B-10	160	159	0.994
Average value			1.005
standard deviation			0.033
coefficient of variation			0.033

**Table 7:** Parameter details of the numerical calculation models and the GFRPP reinforcement softening time.

Model Number	*T*_r1_ (°C)	*T*_0_ (°C)	*T*_center_ (°C)	*r*_silicone_ (mm)	*d* (mm)	*t*_m_ (s)
MH-01	200	25	160	0	7.8	125
MH-02	200	25	160	1.0	7.8	186
MH-03	200	25	160	1.5	7.8	215
MH-04	200	25	160	2.0	7.8	244
MH-05	200	25	160	0	9.8	195
MH-06	200	25	160	1.0	9.8	270
MH-07	200	25	160	1.5	9.8	307
MH-08	200	25	160	2.0	9.8	344
MH-09	200	25	160	0	11.8	280
MH-10	200	25	160	1.0	11.8	372
MH-11	200	25	160	1.5	11.8	416
MH-12	200	25	160	2.0	11.8	459
MH-13	200	25	160	0	15.8	497
MH-14	200	25	160	1.0	15.8	619
MH-15	200	25	160	1.5	15.8	680
MH-16	200	25	160	2.0	15.8	739
MH-17	200	25	160	0	19.8	774
MH-18	200	25	160	1.0	19.8	927
MH-19	200	25	160	1.5	19.8	1007
MH-20	200	25	160	2.0	19.8	1082
MH-21	230	25	160	0	7.8	82
MH-22	230	25	160	1.0	7.8	121
MH-23	230	25	160	1.5	7.8	141
MH-24	230	25	160	2.0	7.8	160
MH-25	230	25	160	0	9.8	128
MH-26	230	25	160	1.0	9.8	177
MH-27	230	25	160	1.5	9.8	201
MH-28	230	25	160	2.0	9.8	225
MH-29	230	25	160	0	11.8	184
MH-30	230	25	160	1.0	11.8	244
MH-31	230	25	160	1.5	11.8	273
MH-32	230	25	160	2.0	11.8	301
MH-33	230	25	160	0	15.8	327
MH-34	230	25	160	1.0	15.8	407
MH-35	230	25	160	1.5	15.8	447
MH-36	230	25	160	2	15.8	485
MH-37	230	25	160	0	19.8	510
MH-38	230	25	160	1.0	19.8	610
MH-39	230	25	160	1.5	19.8	660
MH-40	230	25	160	2	19.8	709
MH-41	260	25	160	0	7.8	62
MH-42	260	25	160	1.0	7.8	92
MH-43	260	25	160	1.5	7.8	107
MH-44	260	25	160	2.0	7.8	122
MH-45	260	25	160	0	9.8	97
MH-46	260	25	160	1.0	9.8	135
MH-47	260	25	160	1.5	9.8	153
MH-48	260	25	160	2.0	9.8	171
MH-49	260	25	160	0	11.8	140
MH-50	260	25	160	1.0	11.8	185
MH-51	260	25	160	1.5	11.8	207
MH-52	260	25	160	2.0	11.8	229
MH-53	260	25	160	0	15.8	249
MH-54	260	25	160	1.0	15.8	310
MH-55	260	25	160	1.5	15.8	340
MH-56	260	25	160	2.0	15.8	369
MH-57	260	25	160	0	19.8	388
MH-58	260	25	160	1.0	19.8	465
MH-59	260	25	160	1.5	19.8	502
MH-60	260	25	160	2.0	19.8	539

**Table 8:** Values of a and b for different GFRPP reinforcement diameters.

GFRPP Reinforcement Diameter *d* (mm)	*d*/9.8	*a*	*b*
7.8	0.80	0.05	0.64
9.8	1.00	0.00	1.00
11.8	1.20	−0.08	1.43
15.8	1.61	−0.30	2.54
19.8	2.02	−0.63	3.94

**Table 9:** Calculation parameters and results.

Model Number	*A*	$\bar{D_{GF}}$	*t*_m_ (s)	*t*_eq_ (s)	*t*_eq_ */t*_m_
MH-01	0.2286	0.0258	125	125	1.000
MH-02	0.2286	0.0258	186	181	0.973
MH-03	0.2286	0.0258	215	211	0.981
MH-04	0.2286	0.0258	244	242	0.992
MH-05	0.2286	0.0258	195	195	1.000
MH-06	0.2286	0.0258	270	268	0.993
MH-07	0.2286	0.0258	307	305	0.993
MH-08	0.2286	0.0258	344	341	0.991
MH-09	0.2286	0.0258	280	280	1.000
MH-10	0.2286	0.0258	372	371	0.997
MH-11	0.2286	0.0258	416	414	0.995
MH-12	0.2286	0.0258	459	455	0.991
MH-13	0.2286	0.0258	497	495	0.996
MH-14	0.2286	0.0258	619	626	1.011
MH-15	0.2286	0.0258	680	681	1.001
MH-16	0.2286	0.0258	739	728	0.985
MH-17	0.2286	0.0258	774	768	0.992
MH-18	0.2286	0.0258	927	944	1.018
MH-19	0.2286	0.0258	1007	1009	1.002
MH-20	0.2286	0.0258	1082	1058	0.978
MH-21	0.3415	0.0319	82	82	1.000
MH-22	0.3415	0.0319	121	118	0.975
MH-23	0.3415	0.0319	141	138	0.979
MH-24	0.3415	0.0319	160	158	0.988
MH-25	0.3415	0.0319	128	127	0.992
MH-26	0.3415	0.0319	177	175	0.989
MH-27	0.3415	0.0319	201	199	0.990
MH-28	0.3415	0.0319	225	223	0.991
MH-29	0.3415	0.0319	184	183	0.995
MH-30	0.3415	0.0319	244	243	0.996
MH-31	0.3415	0.0319	273	271	0.993
MH-32	0.3415	0.0319	301	297	0.987
MH-33	0.3415	0.0319	327	323	0.988
MH-34	0.3415	0.0319	407	409	1.005
MH-35	0.3415	0.0319	447	445	0.996
MH-36	0.3415	0.0319	485	476	0.981
MH-37	0.3415	0.0319	510	502	0.984
MH-38	0.3415	0.0319	610	616	1.010
MH-39	0.3415	0.0319	660	659	0.998
MH-40	0.3415	0.0319	709	691	0.975
MH-41	0.4255	0.0366	62	65	1.048
MH-42	0.4255	0.0366	92	95	1.033
MH-43	0.4255	0.0366	107	110	1.028
MH-44	0.4255	0.0366	122	127	1.041
MH-45	0.4255	0.0366	97	102	1.052
MH-46	0.4255	0.0366	135	140	1.037
MH-47	0.4255	0.0366	153	159	1.039
MH-48	0.4255	0.0366	171	179	1.047
MH-49	0.4255	0.0366	140	147	1.050
MH-50	0.4255	0.0366	185	194	1.049
MH-51	0.4255	0.0366	207	217	1.048
MH-52	0.4255	0.0366	229	238	1.039
MH-53	0.4255	0.0366	249	259	1.040
MH-54	0.4255	0.0366	310	328	1.058
MH-55	0.4255	0.0366	340	357	1.050
MH-56	0.4255	0.0366	369	381	1.033
MH-57	0.4255	0.0366	388	402	1.036
MH-58	0.4255	0.0366	465	494	1.062
MH-59	0.4255	0.0366	502	528	1.052
MH-60	0.4255	0.0366	539	554	1.028
Average value					1.010
standard deviation					0.026
coefficient of variation					0.026

## Data Availability

The original contributions presented in the study are included in the article, further inquiries can be directed to the corresponding author.
